# Diffuse zirkuläre Halsschwellung unklarer Genese

**DOI:** 10.1055/a-2664-1149

**Published:** 2025-08-05

**Authors:** Louis Jansen, Tim Koppen, Helen Abing, Julia Esser, Shachi Jenny Sharma, Nora Wuerdemann, Jens Peter Klußmann, Friedrich Bootz, Arthur Charpentier

**Affiliations:** 1 27182 Klinik und Poliklinik für Hals-Nasen-Ohrenheilkunde, Kopf-Hals-Chirurgie, Universitätsklinikum Köln, Cologne, Germany; 2 39062 Klinik und Poliklinik für Hals-Nasen-Ohrenheilkunde, Kopf- und Halschirurgie, Universitätsklinikum Bonn, Bonn, Germany; 3 27182 Klinik und Poliklinik für Hals-Nasen-Ohrenheilkunde, Kopf- und Halschirurgie, Universitätsklinikum Köln, Cologne, Germany; 4 Klinik für Innere Medizin I, Medizinische Fakultät, Universitätsklinik zu Köln, Köln, Germany

## Anamnese

Eine 30-jährige Patientin wurde aus einem externen Krankenhaus in das interdisziplinäre Notfallzentrum zur kardiologischen Abklärung rezidivierender Synkopen überwiesen. Sie hatte seit einigen Tagen eine langsam voranschreitende, zirkuläre Halsschwellung. Diese war bereits mehrfach aufgetreten, hatte sich aber jedes Mal spontan zurückgebildet. Aufgrund einer unklaren Serositis mit Pleura- und Perikardergüssen sowie einer Gewichtszunahme wurden in den letzten Wochen eine umfangreiche serologische Diagnostik (inkl. Screening rheumatologischer Parameter) und eine 24-stündige Sammelurindiagnostik durchgeführt. Diese ergaben keinen Hinweis auf eine rheumatologische oder nephrologische Ursache. Zudem wurde auswärtig bereits eine Computertomografie (CT) des Thorax durchgeführt, welche die mögliche Ursache der Halsschwellung jedoch nicht eingrenzen konnte. Hier wurden einzig Pleura- und Perikardergüsse sowie etwas vergrößerte mediastinale Lymphknoten beschrieben.

Bei der Erstvorstellung bestanden keine Dyspnoe, Heiserkeit, Dysphagie oder Fieber. Die Patientin berichtete über eine nicht näher bezeichnete Leukämie in der Kindheit, 2 Meningitiden und eine Follikelblutung vor vielen Jahren. Außerdem sei eine Borderline-Störung mit instabiler Persönlichkeitsstörung bekannt, die in der Vergangenheit bereits zu zahlreichen vorangegangenen Hospitalisierungen geführt hatte. Sie nimmt dauerhaft Medikamente, ein atypisches Neuroleptikum und ein Antidepressivum. Vor einem Jahr wurde aufgrund des schlechten Venenstatus und wiederholter Krankenhausaufenthalte die Implantation eines Portkatheters linkspektoral durchgeführt. Es bestanden Medikamentenallergien auf Ketamin, Ibuprofen und Perfalgan.

## Befund und Diagnostik

**Abb. 1:**
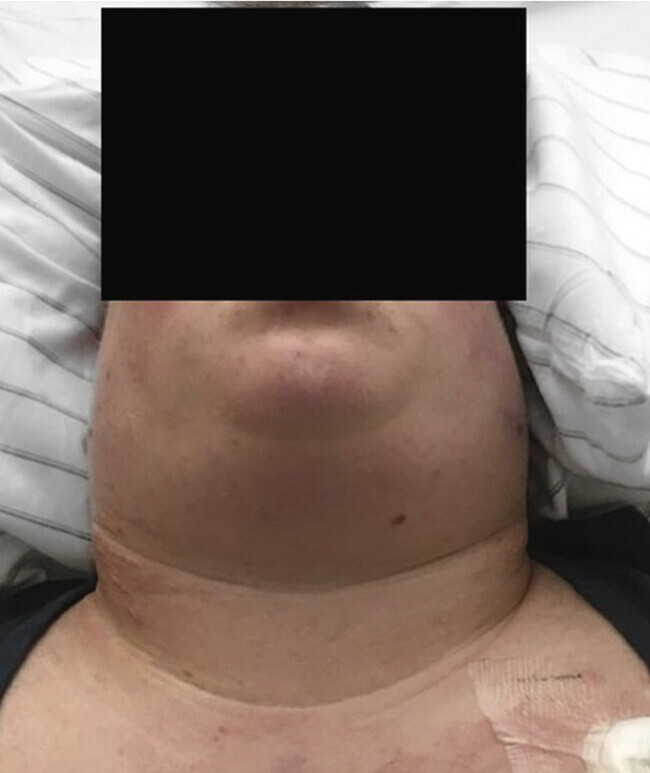
30-jährige Patientin mit ausgeprägter zirkulärer Halsschwellung.

**Abb. 2:**
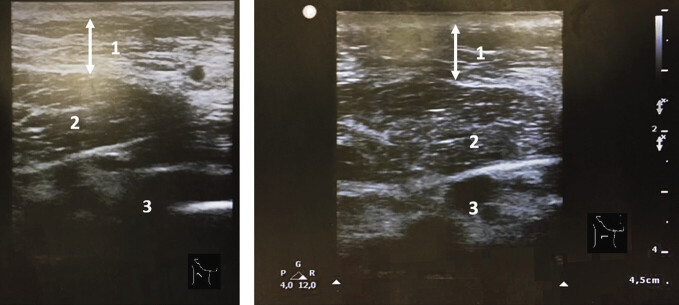
Sonografie der Kopf- und Halsweichteile rechts zervikal Level III. Diffuse Weichteilschwellung (1), Musculus sternocleidomastoideus (2), Halsgefäßnervenscheide mit Vena jugularis interna (3).

Bei einer adipösen Patientin (Body-Mass-Index von 36,8kg/m²) wurde im Rahmen der HNO-ärztlichen Befunderhebung eine ausgeprägte zirkuläre, palpatorisch weiche Halsschwellung ( Abb. 1 ) festgestellt. Die Vitalparameter waren im Normbereich, die pulsoxymetrisch gemessene Sauerstoffsättigung lag bei über 95% unter Raumluft. Die Schwellung war lokal auf den Hals begrenzt ohne Ausbreitung auf Gesicht oder Extremitäten. Die Haut war weder gerötet noch überwärmt. Eine Kieferklemme lag nicht vor. Lippen und Zunge waren unauffällig. Es bestanden keine enoralen oder oropharyngealen Ödeme, Asymmetrien oder Enantheme. Der Speichelfluss war regelhaft. Die flexible Pharynx- und Larynxendoskopie ergab, dass der Pharynx und der Larynx frei waren und die Stimmlippen beidseits beweglich. Die in der Erstuntersuchung durchgeführte B-Mode-Sonografie zeigte eine diffuse subkutane Weichteilschwellung des Halses und eine reaktive zervikale Lymphadenopathie ohne Malignitätskriterien oder Einschmelzungen ( Abb. 2 ). Eine eindrückliche Distension der Vena jugularis war nicht zu beobachten. Die großen Speicheldrüsen und die Schilddrüse waren unauffällig. Es zeigten sich weder ein Schleimhautödem noch ein Exanthem oder Enanthem. Die Familienanamnese, die Eigenanamnese sowie die Triggeranamnese (z.B. Einnahme von ACE-Hemmern) waren unauffällig. Die relevanten laborchemischen Parameter sind in Tab. 1 zusammengefasst.

**Table:** **Tab. 1** Ausgewählte Laborparameter und Referenzwerte bei Aufnahme.

Laborparameter	Wert	Referenzbereich
Leukozyten	13,37g/l	3,9–10,2g/l
C-reaktives Protein (CRP)	94,77mg/l	0–3mg/l
Gesamteiweiß	70,2g/dl	65–83g/dl

## Verlauf, Diagnose und Therapie

Aufgrund der Schwellung wurden initial eine hochdosierte intravenöse Gabe von Prednisolon (250mg) sowie ein H1-Antihistaminikum verabreicht. Eine Besserung trat jedoch nicht ein. Insgesamt zeigte sich kein typisches Bild einer anaphylaktischen Reaktion. Die klinischen Befunde entsprachen auch nicht denen eines hereditären oder nicht hereditären Angioödems. Trotz normaler Konzentration und Aktivität des C1-Esterase-Inhibitors und normaler C4-Komplementspiegel (wie sie z.B. bei einem hereditären Angioödem mit normalen C1-INH-Serumspiegeln auftreten können) wurde die Therapie durch die Gabe eines C1-Esterase-Inhibitorkonzentrats ergänzt. Auch dieser Therapieversuch war erfolglos. Bei nun zunehmender kardiopulmonaler Instabilität bestand die Indikation zur fiberoptischen Intubation und intensivmedizinischen Überwachung der Patientin. Die transthorakale Echokardiografie zeigte einen Perikarderguss, der punktiert wurde. Dieser stellte sich als milchig-trübes Sekret dar. Eine mikrobiologische Untersuchung konnte keinen Erregernachweis erbringen (insb. keine Mykobakterien).

**Abb. 3:**
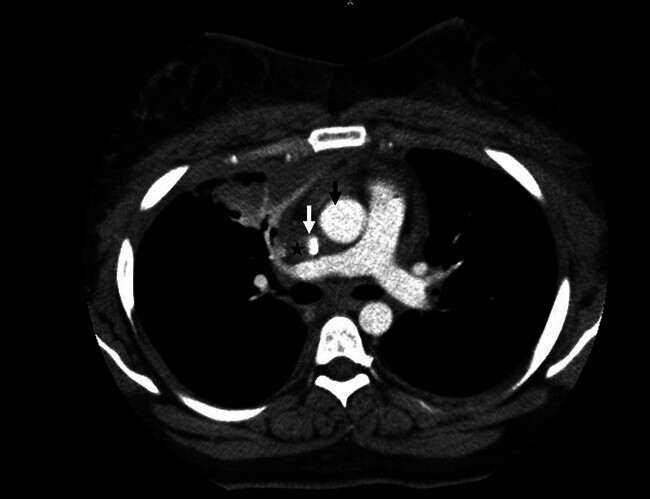
Computertomografie des Thorax, axial, mit Kontrastmittel. Darstellung der regelrecht kontrastierten Aorta ascendens (schwarzer Pfeil), der von Kontrastmittel ausgesparten Vena cava superior (*) und des anflutenden Kontrastmittels im Restlumen der Vena cava superior (weißer Pfeil).

**Abb. 4:**
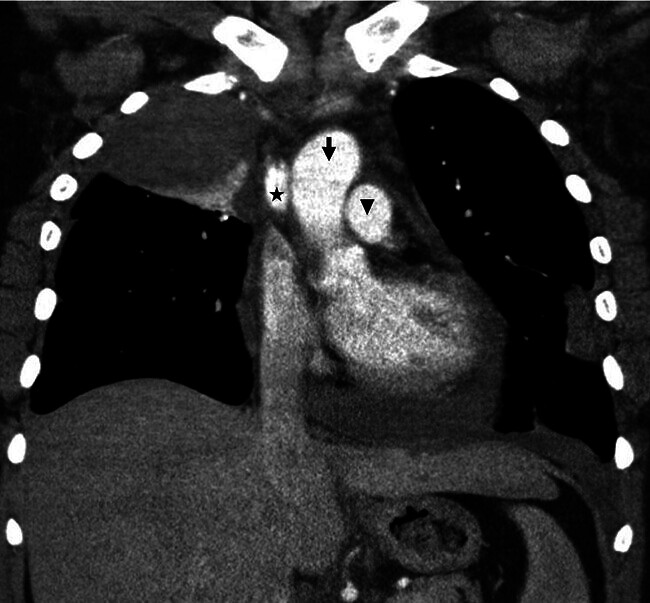
Computertomografie des Thorax, koronar, mit Kontrastmittel. Regelrecht kontrastierte Aorta ascendens (schwarzer Pfeil), regelrecht kontrastierte Pulmonalvene (kurzer schwarzer Pfeil) und Vena cava superior mit Kontrastmittelaussparung (*).

Bei weiterhin unklarer Ätiologie, aber unverändertem klinischem Befund mit generalisierter Schwellung der Halsweichteile inklusive Perikarderguss und Thoraxschmerzen wurde eine erneute CT des Halses und Thorax mit Kontrastmittel (ohne venöse Phase) durchgeführt. Hier wurde erstmalig der Verdacht auf einen Verschluss der Vena cava superior geäußert. Die Vena cava superior zeigte sich partiell verschlossen, zusätzlich zeigten sich eine retrosternale Flüssigkeitsretention und ein Verschluss der V. brachiocephalica links. ( Abb. 3 , Abb. 4 ).

Es erfolgten die umgehende Portexplantation und eine radiologisch kontrollierte Thrombektomie mit interventioneller Implantation zweier Stents in die Vena brachiocephalica beidseits. Am Folgetag zeigten sich in einer Kontrollangiografie ein subtotaler Stentverschluss beidseits, ein Verschluss der Vv. jugulares internae und der Vv. subclaviae beidseits. Deshalb wurde eine radiologisch kontrollierte Thrombektomie mittels Angiojet durchgeführt, begleitet von lokaler Lyse und perkutaner Thrombaspiration. Bei Verdacht auf eine intrainterventionelle Perforation der V. jugularis interna links wurde ein zusätzliches Stenting (Stentgrafts, Viabahn, Gore) derselben durchgeführt. Bereits am Folgetag zeigte sich die Halsschwellung klinisch deutlich regredient. Es kam zu keinen hämodynamisch relevanten Restenosen. Die Patientin wurde nach erfolgreichem Weaning auf die HNO-ärztliche Normalstation übernommen.

## Diskussion

Im vorliegenden Fall wurde die Diagnose einer Port-assoziierten Thrombose mit zeitlicher Verzögerung gestellt. Bei Aufnahme der Patientin lagen bereits laborchemische und bildgebende Befunde vor, weshalb initial von einer Wiederholung der Computertomografie abgesehen wurde. Die Diagnosestellung gestaltete sich zusätzlich herausfordernd, da der zugrunde liegende Auslöser – der Portkatheter – aufgrund der psychiatrischen Grunderkrankung der Patientin erst verspätet in der Anamnese erfasst werden konnte. In der initial durchgeführten Sonografie des Halses hätte retrospektiv eine Distension der Vena jugularis jedoch diskutiert werden können. In der auswärtig durchgeführten Computertomografie des Thorax, welche auch kaudale Teile des Halses miterfasste, zeigte sich jedoch keine signifikante Dilatation der Venae jugulares, weshalb sich nicht umgehend für eine erneute CT entschieden wurde, sondern diese erst in der späteren Differenzialdiagnostik erfolgte. Eine frühzeitige Erwägung und Berücksichtigung eines Vena-cava-superior-Syndroms (VCSS) hätten in diesem Falle möglicherweise die Anzahl diagnostischer Maßnahmen reduziert und die zeitnahe Einleitung einer spezifischen Therapie ermöglicht.

Bei unklaren Schwellungen des Halses müssen anaphylaktische Reaktionen, Angioödeme, entzündliche Geschehen mit Abszedierung, Autoimmunerkrankungen, Lymphgefäß- und Lymphadenopathien sowie benigne und maligne Tumoren und Lipomatosen in die differenzialdiagnostischen Überlegungen einbezogen werden. Größere Hämatome oder Hautemphyseme kommen postoperativ oder posttraumatisch in Betracht. Eine seltene, aber potenziell tödlich verlaufende Differenzialdiagnose ist das Vena-cava-superior-Syndrom 1 .

Dabei handelt es sich um eine Abflussstörung des Versorgungsbereichs der Vena cava superior. Die daraus resultierenden Einschränkungen der venösen Blutzirkulation begünstigen die Thromboseentstehung. Die pathophysiologischen Ursachen sind beschrieben durch die Virchow-Trias 2 . Veränderungen der Gefäßwand, der Blutströmung oder der Blutzusammensetzung fördern eine peripher- oder zentralvenöse Thrombose. VCSS wird durch externe Kompression, interne Obstruktion oder Infiltration der Gefäßwand durch Tumoren verursacht 3 . In den USA kommt es jährlich zu 15000 Fällen. Für Deutschland liegen noch keine Daten vor. Der überwiegende Anteil aller VCSS lässt sich auf mediastinale Malignome zurückführen (70–90%), wobei das kleinzellige Bronchialkarzinom und das mediastinale Non-Hodgkin-Lymphom führend sind 3 . Der zunehmende Einsatz intravenös eingebrachter zentralvenöser Katheter und Schrittmacher führt zu einer erhöhten Anzahl iatrogener VCSS. Betroffene zeigen einen typischen Symptomkomplex aus Husten, konjunktivaler Suffusion, Dysphonie, Dysphagie und Dyspnoe mit Stridor 3 . Seltener treten generalisierte Symptome wie Bewusstseinstrübung, Visusveränderung und Koma auf.

Die obere Einflussstauung führt zu Ödemen der oberen Extremitäten. Zusätzlich imponiert bereits eine Halsvenenstauung oder es sind Kollateralen als Halsvenenkranz erkennbar. Die Stauung lässt sich durch Oberkörperhochlagerung gegebenenfalls entlasten.

Die Diagnose des lebensbedrohlichen VCSS erfolgt über die Computertomografie mit Angiografie des Halses und des Thorax oder über die kontrastmittelgestützte Venografie 4 . Ersteres ist der Goldstandard in der schnell verfügbaren Primärdiagnostik.

Die Therapie der oberen Einflussstauung richtet sich nach den zugrunde liegenden Ursachen und deren Folgen. Wenn eine VCSS durch ein in eine Körpervene eingebrachtes Fremdmaterial verursacht wurde, muss dieses unverzüglich entfernt werden.

Bei tumorbedingten Einflussstauungen kann eine rasch eingeleitete Strahlentherapie oder auch Chemotherapie zu einer Dekompression führen. Manifeste Thrombosen werden antikoaguliert oder thrombolysiert. In akut lebensbedrohlichen Zuständen oder bei fehlendem Erfolg der Thrombolyse ist eine interventionelle Ballondilatation mit Stenteinsatz zwingend erforderlich 4 5 .

**Diagnose: Vena-cava-superior-Syndrom (VCSS)**
